# How plants with hundreds of centromeres crossover

**DOI:** 10.1038/s41477-024-01646-7

**Published:** 2024-03-01

**Authors:** Meng Zhang, André Marques

**Affiliations:** Max Planck Institute for Plant Breeding Research, Cologne, Germany

## Abstract

Beaksedges harbour multiple centromeres in each chromosome, yet crossover distribution is distally biased, like in monocentric species, but with no correlation with (epi)genomic features. This study suggests that synapsis dynamics starting from chromosomal ends is key to the recombination pattern.

## The question

Meiotic recombination occurs during meiosis (the formation of gametes (sperm and egg cells)) in sexually reproducing organisms. It results from the pairing of the two copies of homologous (same) chromosomes and the crossover (exchange) of genetic material between the chromosomes − genes are shuffled around resulting in genetic diversity in the offspring^1^. Centromeres are specialized regions of chromosomes that help chromosomes to divide properly during mitosis (equational division of somatic cells) and meiosis. In most eukaryotes, centromeres are localized to a single specific region of the chromosome, that is, chromosomes have a monocentromere^2^. However, in several animals and plants, such as *Rhynchospora breviuscula* (a beaksedge), centromeres are diffusely distributed along the entire length of the chromosome, forming a structure known as a holocentromere^3^. Previous studies showed that in species with monocentromeres, the frequency and distribution of meiotic recombination events are not random but tightly regulated; for example, chromosomes rarely crossover at and close to centromeres^4^. The fact that hundreds of centromeres are spread all along chromosomes in *Rhynchospora* spp. raises questions about how meiotic recombination is regulated and distributed in holocentric (having chromosomes with holocentromeres) plants.

## The discovery

We used immunostaining to localize some key functional proteins during meiosis and observe chromosomal behaviours in *R. breviuscula*. Because *R. breviuscula* is highly self-incompatible (cannot generate zygotes after self-pollination), to quantify genome-wide crossovers we genotyped male gametes (which carry the results of crossovers and can be obtained in large amounts and easily from pollen) using high-throughput single-cell RNA sequencing. After sequencing thousands of *R. breviuscula* pollen grains, we constructed a recombination landscape for each chromosome. We also performed chromatin immunoprecipitation with sequencing (ChIP−seq) to investigate potential factors that influence recombination patterns, because crossover distribution is typically associated with genetic and epigenetic features. Finally, to infer the existence of the centromere effect (depletion of crossovers near and/or at centromeres), we sequenced the whole genome DNA of 63 plants generated with self-pollination, because single-cell RNA sequencing only sequences genomic regions where transcription occurs.

**Fig. 1|:**
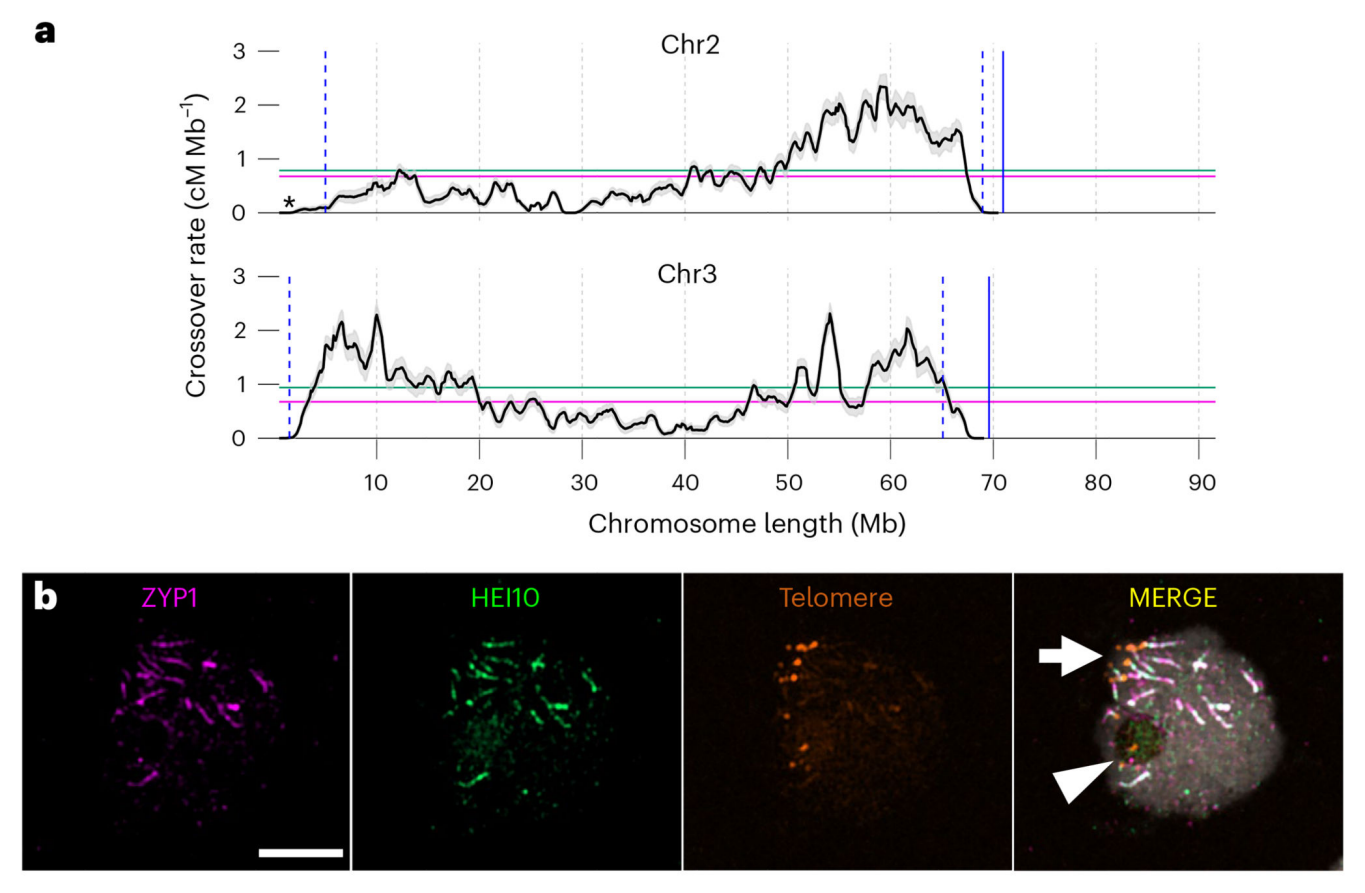
Distally biased crossover distribution in the holocentric plant *R. breviuscula*. **a**, Distribution of the crossover rate in *R. breviuscula* chromosome (chr) 2 and 3. The black line and grey shadowed areas around it indicate the mean and standard deviation, respectively, of the crossover frequency in cM Mb^−1^, centimorgan per megabase. Asterisk indicates the position of ribosomal DNA. Blue dashed lines indicate the start and end of confident crossover rate computation. Blue solid lines indicate chromosomal end. Magenta and green lines indicate: genome-wide and chromosome-wide average crossover rates, respectively. **b**, In the zygotene (the meiotic prophase state when synapsis starts), ZYP1 lines (a protein that indicates synapsis) are first seen close to the ‘bouquet’ configuration (arrow); similarly, the first HEI10 (a protein that stabilizes recombination intermediate structures) signals are seen on synapsed chromosomes, whereas some telomeres localize to the nucleolus (arrowhead) and lack the ZYP1 and HEI10 signals. Scale bar, 5 µm. (c) 2024, Castellani, M., CCBY 4.0.

Cytological observations revealed that the behaviour of *R. breviuscula* chromosomes was similar to that of chromosomes in monocentric plants (for example, *Arabidopsis thaliana*). However, in *R. breviuscula* crossover frequency is distally biased, that is, crossover occurs more frequently at the chromosome ends (telomeres) than in other regions, as it is in monocentric species, despite almost evenly distributed (epi) genetic features and chromosomal compartments (Fig. 1a). Moreover, crossovers are abolished inside centromeric units but not in their proximity, indicating the absence of a canonical centromere effect. We also found evidence for interference between crossovers, suggesting that the positioning of one crossover can affect the likelihood of another one forming nearby. Further analysis of temporal and spatial chromosomal behaviours showed that telomeric regions that paired and formed a synapsis (close association) early during meiosis had high recombination rates, whereas telomeres that formed a synapsis late during the process and central chromosomal regions had low recombination rates (Fig. 1b). Our findings suggest that the primary influence on the broad crossover distribution in *R. breviuscula* is the mechanistic features of meiotic pairing and synapsis, rather than (epi)genomic features and centromere organization.

## Future directions

Our study sheds light on the primary drivers of crossover patterning in holocentric plants and on the evolution of meiotic recombination in eukaryotes. The finding that telomere-led pairing and synapsis are the primary forces determining the distally biased recombination landscape challenges the previous notion that crossover distribution is primarily influenced by chromosome compartmentalization and centromere organization.

However, there are still unanswered questions, including the molecular mechanisms underlying telomere-led pairing and synapsis, the effects of holocentromeres in meiotic chromosome segregation and the factors that regulate crossover interference. By addressing these challenges, we can gain a deeper understanding of the fundamental processes that shape genetic diversity. Meiotic recombination shows immense variability among species, so it is important to decipher the molecular mechanisms that govern interspecific recombination patterns and their implications for phenotypic traits.

## Expert Opinion

“This article reports the crossover patterning in the holocentric plant *R. breviuscula*. This is a very interesting study, because in most species, centromeres strongly affect the overall distribution and frequency of crossovers, and these phenomena can extend by several megabases in plants with large chromosomes. How crossovers are distributed in a holocentric plant is therefore of primary importance to better understand the control of crossover localization.”

An anonymous reviewer.
